# Angle-closure glaucoma in a patient with the nanophthalmos-ocular cystinosis-foveoschisis-pigmentary retinal dystrophy complex

**DOI:** 10.1186/1471-2415-12-23

**Published:** 2012-07-16

**Authors:** Kenan Sonmez, Pehmen Y Ozcan

**Affiliations:** 1 Ulucanlar Eye Training and Research Hospital, Third Eye Clinic, Ankara, Turkey

**Keywords:** Angle-closure glaucoma, Nanophthalmos, Ocular cystinosis, Foveoschisis, Pigmentary retinal dystrophy

## Abstract

**Background:**

To report clinical features of bilateral angle-closure glaucoma in a patient with nanophthalmic eyes associated with ocular cystinosis, foveoschisis and pigmentary retinal dystrophy. This is probably the first published report of the possible association of all these five entities in the same patient.

**Case presentation:**

A 50-year-old white male was referred for uncontrolled glaucoma in both eyes. He was previously diagnosed with angle-closure glaucoma in association with ocular cystinosis. Ocular examination revealed high hyperopia (**+**13.5 OD and +14 OS diopters) with reduced axial length (16.27 mm OD and 15.93 mm OS). Despite being on 3 topical medications, his IOP measured 37 mmHg OD and 35 mm Hg OS. Slit-lamp biomicroscopy showed refractile, polychromatic crystalline deposits throughout the cornea and conjunctiva in both eyes. Gonioscopy revealed an extremely narrow angle with peripheral anterior synechiae (PAS). Anterior chamber depths were shallow**.** Fundus examination disclosed punctate hypopigmentation of the retinal pigment epithelium mainly at the posterior pole. Optical coherence tomography showed foveal schisis appearing as small retinal cysts. The patient did not display any systemic abnormalities.

**Conclusions:**

This case brings into discussion a new clinical entity of angle closure glaucoma in nanophthalmos accompanied by ocular cystinosis-foveoschisis-pigmentary retinal dystrophy complex.

## Background

Nanophthalmos is a rare ophthalmic entity presenting with potential sight-threatening complications such as glaucoma. The condition may occur in isolation or as part of a rare syndrome. The association of nanophthalmos and pigmentary retinal dystrophy with or without foveoschisis or optic disc drusen has been described in the literature after its first description as a new syndrome in 1985 [1,2]. An autosomal recessive inheritance pattern was described for this association [3]. Recently, membrane frizzled-related protein (MFRP) mutations were identified in patients with nanophthalmos associated with or without retinal disfunction [2]. However it is not clear why some patients with MFRP mutations have isolated nanophthalmos and the others present with the complex of nanophthalmos-retinitis pigmentosa-foveoschisis-optic disc drusen.

Here, we report a case of angle closure glaucoma in nanophthalmos accompanied by ocular cystinosis, foveoschisis and pigmentary retinal dystrophy. To the best of our knowledge, this is the first report that describes angle closure glaucoma in nanophthalmic eye associated with ocular cystinosis, foveoschisis and pigmentary retinal dystrophy.

## Case Presentation

A 50-year-old white man with a history of progressive vision loss and elevated intraocular pressure (IOP) in both eyes was referred to our hospital. He was previously diagnosed with angle-closure glaucoma in association with ocular cystinosis and was on three topical antiglaucoma therapy. Although the leukocyte cystine level was not measured at that time, conjunctival biopsy was reported to support the diagnosis of cystinosis. He underwent comprehensive systemic and ocular examination, and laboratory investigations. His family history was negative for cystinosis and nanophthalmos**.**

**Figure 1:**
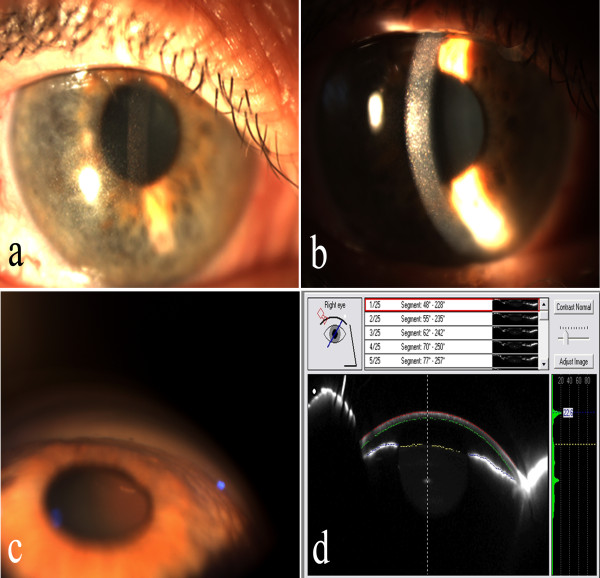
**a) Slit-lamp biomicroscopy of the right eye demostrating characteristic refractile corneal cystine crystals throughout the cornea. b**) Narrow beam slit-lamp biomicroscopy shows that crystals are located throughout the corneal epithelium and entire stromal layer. **c**) Gonioscopic examination reveals a narrow angle with peripheral anterior synechiae in the right eye. **d**) Allegro Oculyzer image of the right eye shows a shallow peripheral anterior chamber with narrow angle.

**Figure 2:**
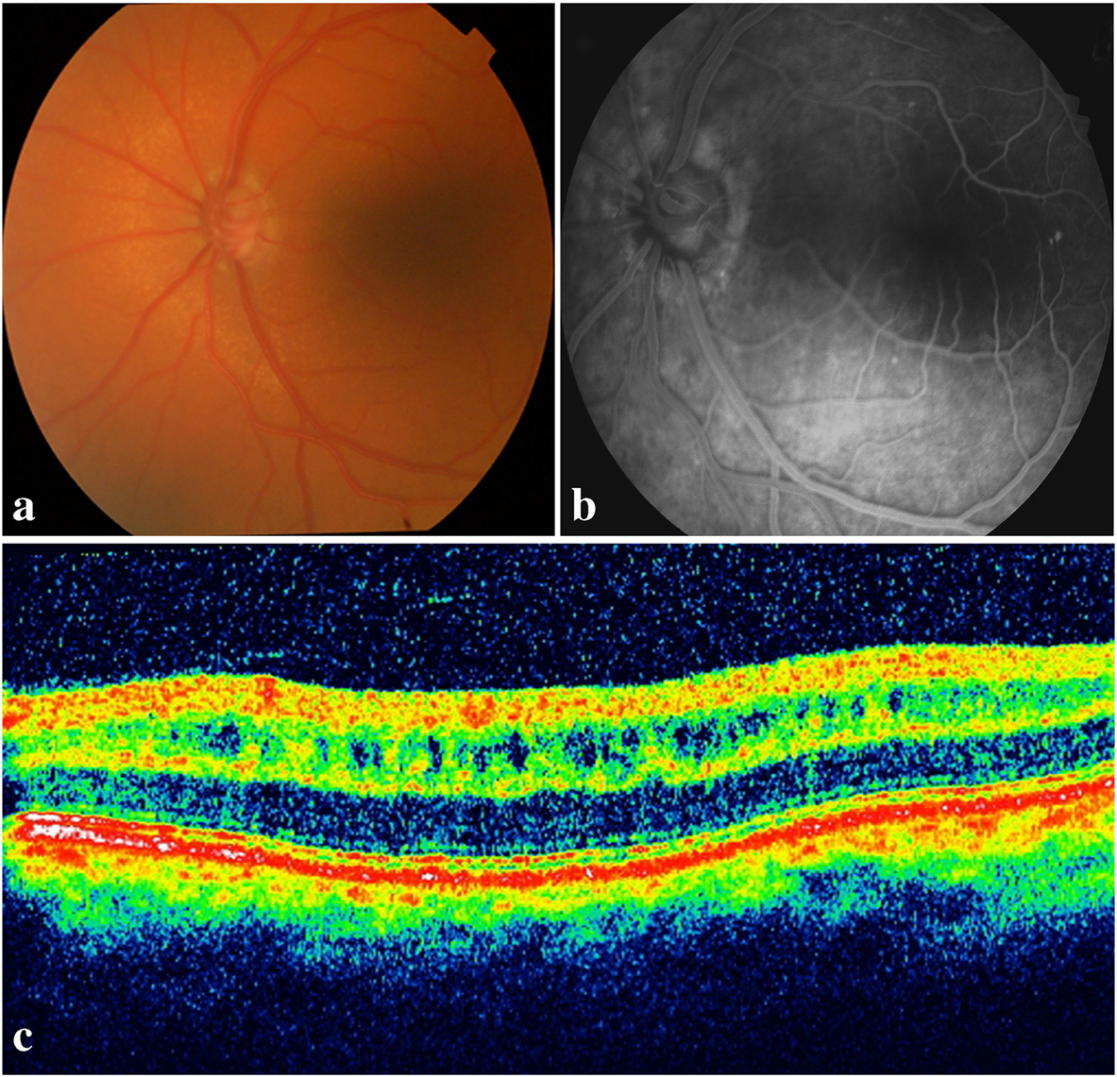
**a**) **Fundus photograph of the left eye reveals round white flecks at the level of the retinal pigment epithelium. b**) Fluorescein angiography shows transmission defects corresponding to observed areas of RPE mottling. **c**) OCT image of the left eye shows increased retinal thickness due to cystoid changes in a schisis-like pattern at the inner nuclear layer of the macula.

On examination, his visual acuity was 20/400 OU with hyperopic correction of **+**13.5 OD and +14 diopters OS. Despite three topical medications, his IOP measured 37 mmHg OD and 35 mm Hg OS and his cup-to-disc ratio was 0.85 OD and 0.8 OS. Slit-lamp biomicroscopy revealed bilateral refractile, polychromatic crystalline deposits in cornea and conjunctiva (Figure 1a and 1b). Gonioscopy revealed an extremely narrow angle with peripheral anterior synechiae (PAS) occupying approximately 50% of each angle (Figure 1c). Both anterior chambers were quite shallow. Anterior chamber depths measured by Allegro Oculyzer were 1.64 mm in the right eye and 1.55 mm in the left eye. Central corneal thickness was 509 μm OD and 506 μm OS. Corneal diameters were 9.5 mm in both eyes. Anterior chamber angle measured with Allegro Oculyzer were 22.2° on inferotemporal side of right eye and 19.1° on the inferotemporal side of left eye (Figure 1d). Ultrasound biomicroscopy (UBM) showed that the angle in both eyes was narrowed 360 degrees circumferentially without an evidence of plateau iris formation and uveal effusion. On A- Scan biometry, the axial lengths were 16.27 mm OD and 15.93 mm OS, with a lens thickness of 4.82 mm and 5.19 mm in the right and left eyes, respectively. Fundus examination revealed hyperopic disc with mild pallor, and areas of stippled and punctate hypopigmentation of the retinal pigment epithelium (RPE) at the midpheriphery and around the optic disc in both eyes (Figure 2a). Fluorescein angiography (FA) showed choroidal transmission hyperfluorescence corresponding to RPE atrophy (Figure 2b). Optical coherence tomography (OCT) imaging also revealed cystoid changes in a schisis-like pattern which was not detectable clinically (Figure 2c). The electroretinography showed that both rod and cone responses were affected and the b-waves were reduced in amplitude with mildly delayed implicit time. The serum creatinine was 0.7 mg/dL, with normal electrolytes and blood counts. The urinalysis was normal. Leukocyte cystine level determined using the cystine-binding protein method was 1.1 nmol half-cystine/mg protein. No medical evidence of renal tubular or glomerular damage was found. Bilateral Nd:YAG laser iridotomy was performed in both eyes, but the angle opened minimally and the IOP remained unchanged. Patient refused further operations despite recommendations.

## Conclusions

Angle-closure glaucoma has been described in association with metabolic or ocular disorders like cystinosis and nanophthalmos. Nanophthalmos is a rare, bilateral and sight threatening disease characterized by hyperopia, short axial length, small corneal diameter, thickened sclera, shallow anterior chamber and narrow angle [4]. Patients with nanophthalmos have risk for developing angle-closure glaucoma by the fourth to sixth decade of life due to the progressive shallowing of the anterior chamber and narrowing of the angle [5]. Posterior pushing mechanism leading to pupillary block and peripheral anterior synechiae formation is the most common cause of angle closure glaucoma in nanophthalmic eyes.

Cystinosis is a rare autosomal recessive metabolic disorder characterized by intracellular accumulation of cystine, the disulfide of amino acid cysteine [6]. The responsible gene, CTNS, encodes cystinosin, that transports cystine out of the lysosome [7]. As a result of deficient or absent cystinosin, cystine accumulates within lysosomes and forms crystals in many tissuses, such as kidneys, bone marrow, pancreas, muscle, brain and eye [6]. Based on the age at onset and severity of the symptoms, three clinical forms of cystinosis including infantile nephropathic cystinosis, intermediate cystinosis and ocular cystinosis were described [8]. Ocular cystinosis presents only with ophthalmic symptoms and never exhibit systemic manifestations [6]. Patients with nephropathic cystinosis have leukocyte cystine levels of 5 to 23 nmol of half-cystine/milligram of protein, whereas patients with ocular cystinosis have values of 1 to 3 nmol of half-cystine/milligram of protein [6]. Our patient did not display any systemic abnormalities and leukocyte cystine level (1.1 nmol half-cystine/mg protein ) was consistent with ocular cystinosis.

The glaucoma may be the result of angle closure with pupillary block due to posterior synechiae in cystinosis patients. Iris, ciliary body or angle abnormalities may also contribute to the development of glaucoma in patients with cystinosis [9]. Mungan et al. [9] reported of ocular UBM findings which revealed the ciliary body configuration similar to the findings in plateau iris in patients with cystinosis. They did not observe any evidence of increased iris thickness, posterior synechiae, or anterior iris bowing. However, in the present study, UBM revealed 360 degrees circumferential narrowing of angle without an evidence of plateau iris**-**like configuration and uveal effusion in both eyes. In addition, gonioscopy revealed an extremely narrow angle with peripheral anterior synechiae occupying approximately 50% of each angle.

Crystal accumulation in the conjunctiva and cornea, and pigmentary retinopathy are originally the most commonly described ophthalmic manifestations of cystinosis. Although patients with ocular cystinosis do not exhibit a retinal pigment abnormality, posterior segment involvement associated with retinal degeneration have been described in infantile nephropathic cystinosis [10]. The most common retinal finding in these eyes was patches of depigmentation in the periphery with pigmentary mottling [10]. However, in the present study, areas of stippled and punctate hypopigmentation of RPE without the presence of hiperpigmentation were observed at posterior pole and around the optic disc. Electrophysiological evaluation revealed a cone**-**rod pattern of retinal dysfunction. Our patient exhibited localized foveal schisis demonstrated by OCT. Similar retinal findings have been described in association with nanophtalmos [2]. Zenteno et al. [2] showed alterations of the RPE with cystic changes very similar to those described in our patients in their study and they described the clinical feature of a new syndrome presenting with nanophthalmos-retinitis pigmentosa-foveoschisis-optic disc drusen. While not noted in their patients, angle closure glaucoma and ocular cystinosis were observed in our case. However, optic disc drusen was not detected in our patient.

In conclusion, this case report brings into discussion a new clinical entity of angle closure glaucoma in nanophthalmia accompanied by ocular cystinosis-foveoschisis-pigmentary retinal dystrophy complex. Further study of additional subjects with this complex would help to clarify whether these disorders is simply an unusual coexistence of unrelated pathologies or a new and distinct pathological entity**.**

## Consent

Written informed consent was obtained from the patient for publication of this case report and any accompanying images.

## Competing interests

The authors declare that they have no competing interests.

## Authors’ contributions

All authors participated in conception and design of the case report, and acquisition and interpretation of data. KS also performed manuscript writing. Both authors read and approved the final manuscript.

## Pre-publication history

The pre-publication history for this paper can be accessed here:

http://www.biomedcentral.com/1471-2415/12/23/prepub
